# *Aronia melanocarpa* Prevents Alcohol-Induced Chronic Liver Injury via Regulation of Nrf2 Signaling in C57BL/6 Mice

**DOI:** 10.1155/2020/4054520

**Published:** 2020-01-08

**Authors:** Zhuqian Wang, Yange Liu, Xuyu Zhao, Shuyan Liu, Yang Liu, Di Wang

**Affiliations:** ^1^Engineering Research Center of Chinese Ministry of Education for Edible and Medicinal Fungi, Jilin Agricultural University, Changchun 130118, China; ^2^School of Life Sciences, Jilin University, Changchun 130012, China; ^3^School of Basic Medical Sciences, Nanchang University, Nanchang 330038, China

## Abstract

*Aronia melanocarpa* (AM), which is rich in anthocyanins and procyanidins, has been reported to exert antioxidative and anti-inflammatory effects. This study aimed to systematically analyze the components of AM and explore its effects on alcohol-induced chronic liver injury in mice. A component analysis of AM revealed 17 types of fatty acids, 17 types of amino acids, 8 types of minerals, and 3 types of nucleotides. Chronic alcohol-induced liver injury was established in mice via gradient alcohol feeding over a period of 6 months, with test groups orally receiving AM in the last 6 weeks. AM administration yielded potential hepatoprotective effects by alleviating weight gain and changes in organ indexes, decreasing the ratio of alanine aminotransferase/aspartate aminotransferase, reducing lipid peroxidation, enhancing antioxidant activities, decreasing oxidation-related factor levels, and regulating inflammatory cytokine levels. Histological analyses suggest that AM treatment markedly prevented organ damage in alcohol-exposed mice. Furthermore, AM activated nuclear factor erythroid 2-like 2 (Nrf2) by downregulating the expression of Kelch-like ECH-associated protein 1, resulting in elevated downstream antioxidative enzyme levels. AM activated Nrf2 via modulation of the phosphatidylinositol-3-hydroxykinase/protein kinase B signaling pathway. Altogether, AM prevented alcohol-induced liver injury, potentially by suppressing oxidative stress via the Nrf2 signaling pathway.

## 1. Introduction

Alcoholic liver disease (ALD) is a chronic disease worldwide and is associated with increasing mortality rates [1]. In the early stages, ALD typically manifests as steatosis superimposed by an inflammatory infiltrate and progresses to fibrosis or cirrhosis with continued alcohol intake [2]. Excessive drinking can cause alcoholic fatty liver within 2 or 3 weeks and may have further effects on the immune system [3]. Alcohol consumption is also highly correlated with the progression of alcoholic fatty liver [4], causes liver damage, and helps to enhance the production of proinflammatory cytokines and chemokines [5, 6], which can enhance the concentration of macrophages and neutrophils for promoting the inflammation response [7]. Long-termed alcohol consumption causes dysfunction within the mitochondrial electron transport chain, resulting in the overgeneration of ROS [8, 9]. Furthermore, dyslipidemia resulting from alcohol consumption elicits oxidative and inflammatory responses of varying degrees [10]. Cells possess evolutionarily conserved defensive mechanisms against oxidative stress, including the activation of nuclear factor erythroid 2-like 2 (Nrf2) [11]. The activation of Nrf2-mediated anti-inflammatory pathways is considered an effective way to eliminate excessive ROS [12].

The treatment options for ALD have not changed in the last four decades. Abstinence remains the most effective form of treatment when supported by nutrition and steroids [13]. Limited treatment options are available for patients who are steroid nonresponders or have contraindications to steroid usage (e.g., upper gastrointestinal bleeds, impaired renal functions, and sepsis) [14]. Although corticosteroids (CS) remain a mainstay of treatment for severe alcoholic hepatitis, these drugs are associated with significant risks such as infection, especially in this population of patients [15]. Moreover, the safety of clinical agents still requires rigorous evaluation. Drugs such as metadoxine, an effective drug for hepatic antilipid peroxidation, may induce peripheral neuropathy in patients after long-term use or large dose administration [16]. Thus, safe and effective alternative therapeutic regimens for ALD are urgently required.

Various natural products exhibit strong capacities to scavenge free radicals and exert anti-inflammatory effects. These products are thus prime candidates for the prevention and/or treatment of alcoholic liver disease [17]. *Aronia melanocarpa*, also known as black chokeberry, is reported to be rich in anthocyanins and procyanidins [18]. *A. melanocarpa* is native to Russia and the midwestern United States but has been planted throughout northeastern Asia in regions such as Liaoning and Jilin Provinces in China [19]. *A. melanocarpa* and its metabolites, particularly procyanidins, exert antioxidative properties [20]. The anti-inflammatory effects of a crude extract of *A. melanocarpa* calyx have been confirmed in a rat model of lipopolysaccharide-induced uveitis [21]. Furthermore, the anthocyanin flavonoids produced by *A. melanocarpa* exerted a significant inhibitory effect on pancreatic swelling in a model of acute experimental pancreatitis [22]. Our group previously reported the ameliorating effects of *A. melanocarpa* fruit (AM) on gout and hyperuricemia in mice and rats via the regulation of redox imbalance [23]. These studies demonstrate that AM acts as a powerful antioxidant and anti-inflammatory agent.

In this study, we proposed that AM could alleviate the alcoholic liver damage caused by long-term alcohol intake by inhibiting oxidative stress and reducing inflammation. Our findings from a C57BL/6 mouse model of alcohol-induced chronic liver injury confirm that AM prevents alcohol-induced liver injury by suppressing oxidative stress via the Nrf2 signaling pathway.

## 2. Materials and Methods

### 2.1. AM Preparation and Composition Analysis

Fresh AM was obtained from Jilin Beijia Limited Company (Jilin, China) and homogenized.

#### 2.1.1. Main Component Analysis

As per previous studies, the total protein, total sugar, reducing sugar, alkaloid, flavonoids, and total ash content were detected in AM using the Kjeldahl method [24], the 3,5-dinitrosalicylic acid colorimetric method [25], the phenol-sulfuric acid method [26], bromothymol blue colorimetry [27], the sodium nitrite-aluminum nitrate method [28], and the ashing method [29], respectively.

#### 2.1.2. Amino Acid Analysis

AM samples were hydrolyzed at 110°C for 24 h and then transferred to colorimetric tubes. The supernatants were removed, dried, and dissolved in 0.02 mol/L HCL. Five hundred microliters of the mixture were reacted with 250 *μ*L of 0.1 mol/L phenyl isothiocyanate acetonitrile and 250 *μ*L of 1 mol/L triethylamine acetonitrile for 1 h. After mixing this solution with 2 mL of n-hexane, the separated lower layer was collected and passed through a 0.45 *μ*m organic film. Amino acids were analyzed quantitatively using liquid chromatography (1260, Agilent, Santa Clara, CA, USA).

#### 2.1.3. Fatty Acid Analysis

A 5% KOH-methanol solution was added to the ME powder, placed in a 60°C water bath for 30 min, and then mixed with 14% boron trifluoride-methanol solution at 85°C for 30 min. The samples were then mixed with n-hexane and the levels of fatty acids analyzed using a gas chromatograph (7890A, Agilent).

#### 2.1.4. Nucleotide Analysis

Components were extracted from AM using double distilled water at 50°C for 3 h, followed by centrifugation at 3500 rpm for 10 min. The nucleotides were analyzed using a high-performance liquid chromatography (HPLC) system (APS80-16, AUPOS, Henan, China) equipped with a UV detector (LC-20AD, Shimadzu, Kyoto, Japan) and a C18 column (4.6 mm × 250 mm × 5 *μ*m, 880975-902, Agilent). The mobile phase comprised 5% methanol and 95% (50 mM) NaH_2_PO_4_ at 30°C and a rate of 1 mL/min. Adenosine monophosphate and uridine monophosphate were detected at 254 nm, and cytidine monophosphate was detected at 260 nm.

#### 2.1.5. Anthocyanidin Analysis

AM was ultrasonically extracted for 30 min in a hydrolysis tube and then hydrolyzed in a boiling water bath for 1 h. The samples were passed through a 0.45 *μ*m aqueous phase filter and analyzed using an HPLC system equipped with a UV detector (LC-20AD, Shimadzu, Japan) and a C18 column (4.6 mm × 250 mm × 5 *μ*m, 880975-902, Agilent, USA). The mobile phases included a 1% formic acid-water solution and 1% formic acid acetonitrile solution. The absorbance was measured at a wavelength of 530 nm.

#### 2.1.6. Minerals

AM was pretreated with hydrogen nitrate for 30 min at a temperature of 110°C and an atmospheric pressure of 30 atm. Subsequently, the mineral levels, including potassium (K), sodium (Na), calcium (Ca), magnesium (Mg), iron (Fe), zinc (Zn), selenium (Se), manganese (Mn), chromium (Cr), copper (Cu), lead (Pb), mercury (Hg), arsenic (As), and cadmium (Cd), were determined using inductively coupled plasma optical emission spectroscopy (iCAPQ, Thermo Fisher Scientific, Waltham, MA, USA).

### 2.2. Alcohol-Induced Chronic Liver Injury Induction in Mice

Animal care and experimental protocols were approved by the Institutional Animal Ethics Committee of Jilin University (2018SY0501). Fifty healthy male C57BL/6 mice (8 weeks old, weight: 18–22 g; SYXK (JI)2014-0013; Liaoning Changsheng Biotechnology Co., Ltd., Liaoning, China) were housed under the following conditions: temperature of 23°C ± 1°C, relative humidity of 55%, a 12 h light/12 h dark cycle (lights on 7:00–19:00 h), and standard mouse food pellets and tap water *ad libitum*. The diet composition is as follows: ≤10% moisture, ≥18% crude protein, ≥4% crude fat, ≥5% crude fiber, ≥4% mineral, 1.0-1.8% calcium, and 0.6-1.2% phosphorus.

**Figure 1:**
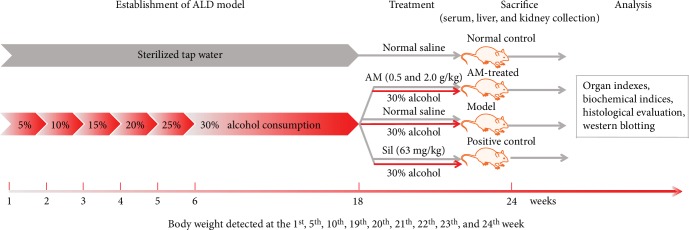
The schedule of the experimental protocol and drug administration. AM: *Aronia melanocarpa*; Sil: silybin.

Mice were randomly separated into five groups (*n* = 10/group) and were intragastrically administered 10 mL/kg normal saline (normal control and model mice) or 63 mg/kg of silymarin (Sil; Madaus AG, Cologne, Germany; positive control mice) [30], as well as 0.5 or 2.0 g/kg of AM at 4:00 pm once per day for 24 weeks. During the first week, all but the control mice received drinking water containing 5% alcohol in sterilized tap water [31]. Gradually, the alcohol content was increased from 5% (*v*/*v*) to 30% (*v*/*v*), with a 5% increase each week [32]. All mice except those in the normal control group then received 30% alcohol until week 18. The body weights were monitored throughout the experimental period (Figure 1).

### 2.3. Blood and Liver Sample Collection

After the final treatment, the mice were fasted overnight and blood samples were collected from the caudal vein. All mice were then anesthetized and sacrificed. The liver, kidney, heart, and spleen were weighed on an electronic balance and stored immediately at -80°C.

### 2.4. Histological Evaluation

Tissues were fixed in a solution of 4% paraformaldehyde in 0.1 M phosphate buffer as described previously [33], dehydrated in gradient alcohol, and embedded in paraffin. Five-micrometer sections were prepared and stained with hematoxylin and eosin (H&E) for histological evaluation.

Kidney tissues were incubated in 0.1% periodic acid for 10 min, washed under running double distilled (D.D.) water for 1 min, and immersed in Schiff's reagent for 15 min. After washing with D.D. water for 3 min, the samples were counterstained with Mayer's hematoxylin for 2 min. After another 3 min wash, the samples were dehydrated twice in 96% alcohol. Finally, the samples were cleared in xylene and mounted with Entellan Neutral gum.

The frozen liver tissue sections were warmed to room temperature for 10 min, then put in D.D. water for cleaning and immersed in sixty percent of isopropyl alcohol solution. After washing with isopropyl alcohol solution, put in Oil red O staining solution, dye for 10-15 minutes, and immerse in sixty percent of isopropyl alcohol solution again for color separation. After immersing, wash tissue twice in D.D. water and with tap water for five to ten minutes during the interval. Finally, sealed with glycerin gelatin.

All stained slides were observed using an inverted microscope (400x) (IX73, Olympus, Tokyo, Japan).

### 2.5. Biochemical Assays

A portion of the liver from each mouse was homogenized with physiological saline on ice. The levels of aspartate aminotransferase (AST; CK-E90386M), alanine aminotransferase (ALT; CK-E90314M), high-density lipoprotein (HDL; CK-E91912M), low-density lipoprotein (LDL; CK-E91911M), triglyceride (TG; CK-E91733M), and total cholesterol (TC; CK-E91839M) in the serum and liver and the hepatic levels of acetoacetyl-CoA synthetase (AACS; CK-E20547M), ROS (CK-E91516M), malondialdehyde (MDA; CK-E20347M), superoxide dismutase (SOD; CK-E20348M), catalase (CAT; CK-E92636M), glutathione peroxidase (GSH-Px; CK-E92669M), amyloid P component (APCS; CK-E20557M), cytochrome P4508B1 (CYP8B1; CK-E20569M), coenzyme 3 (CoQ3; CK-E20565M), microsomal glutathione S-transferase 3 (MGST3; CK-E20553M), thioesterase 4 (THEM4; CK-E20548M), interleukin-2 (IL-2; CK-E41733M), IL-4 (CK-E41732M), IL-6 (CK-E41731M), fibrinogen-like protein 1 (FGL1; CK-E20558M), and fibrinogen *γ* (FGG; CK-E20559M) were detected using enzyme-linked immunosorbent assay (ELISA) kits (Shanghai Yuanye Biological Technology Co., Ltd., Shanghai, China) according to the manufacturer's instructions.

### 2.6. Western Blotting

A portion of the liver from each mouse was homogenized in cell lysis buffer containing 1% protease inhibitor cocktail (Sigma-Aldrich, St. Louis, MO, USA), 2% phenylmethanesulfonyl fluoride (Sigma-Aldrich, St. Louis, MO, USA), and 97% RIPA (Sigma-Aldrich, St. Louis, MO, USA). For detecting the expression levels of Nrf2 in the nucleus of liver tissue, the liver tissues were homogenized using the Cytoplasmic Extraction Reagent I (Thermo, Rockford, USA) following with the incubation of the Cytoplasmic Extraction Reagent II (Thermo, Rockford, USA) for 10 min to obtain the cytoplasmic protein extraction, and then the collected precipitate was incubated with a Nuclear Extraction Reagent (Thermo, Rockford, USA) for another 40 minutes to obtain nuclear protein. The total protein concentrations were measured using a bicinchoninic acid (BCA) protein assay kit (BCA; Merck Millipore, Burlington, Massachusetts, USA). Forty micrograms of protein per sample were separated on a 12% polyacrylamide gel and transferred to a 0.45 *μ*m polyvinylidene fluoride membrane (PVDF; Merck Millipore, Burlington, Massachusetts, USA). After blocking with 5% bovine serum albumin (BSA; Sigma-Aldrich, St. Louis, MO, USA) at 4°C for 4 h, the membrane was incubated with primary antibody (1 : 1000 dilution) against glyceraldehyde-3-phosphate dehydrogenase (GAPDH; ABS16), total-signal transducer and activator of transcription 3 (T-Stat3; 06-596) (Merck Millipore, Burlington, Massachusetts, USA), beta-actin (*β*-actin; sc-47778) (Santa Cruz, 2145 Delaware Ave, USA), phosphor- (P-) Stat3 (cstD3A7), P-phosphatidylinositol-3-hydroxykinase (PI3K; cst4228s), T-PI3K (cst4292s; Cell Signaling Technology, Boston, MA, USA), P-protein kinase B (P-Akt) (ab108266), T-Akt (ab200195), Kelch-like ECH-associated protein 1 (Keap1; ab150654), Nrf2 (ab89443), heme oxygenase-1 (HO-1; ab137749), SOD-2 (ab13533) (Abcam, Cambridge Science Park, UK), HO-2 (bs-1238R), SOD-1 (bs-10216R), and lamin B (bs-1840R) (Bioss Antibodies Biotechnology Co., Ltd., Beijing, China) overnight at 4°C. The membrane was then washed with PBS containing 0.05% Tween-20 and incubated with anti-mouse (IH-0031) and anti-rabbit (IH-0011) (Dingguo Biotechnology Co., Ltd., Beijing, China) horseradish peroxidase-conjugated secondary antibodies for 4 h at 4°C. After three washes, the target proteins were visualized using an Immobilon Western Chemiluminescent HRP substrate (WBKLS0500; Merck Millipore, Burlington, Massachusetts, USA) and a gel imaging system (Biodoc IT2 315, Analytik Jena, Jena, Germany). The bands were quantitated using Image J analysis software, version 1.46 (National Institutes of Health, Bethesda, MD, USA).

### 2.7. Statistical Analysis

Data are expressed as means ± standard errors of the means. The post hoc Dunn's multiple comparison test was performed using SPSS 16.0 software (IBM Corp., Armonk, NY, USA) and analyzed using a one-way analysis of variance (ANOVA). Significance was defined as a *p* value <0.05.

## 3. Results

### 3.1. Composition of AM

The composition of AM was 75.96% total sugar (including 29.3% reducing sugar), 6.61% total protein, 0.021% flavonoids, and 0.065% alkaloids (Table 1). Among the 35 types of fatty acids, 17 were not detected (Table 1). AM also contained 17 types of amino acids, 8 minerals, and 3 nucleotides (Table 1). Regarding the most important components of AM, 3140.45 mg/kg of cyanidin, 24.43 mg/kg of delphinidin, and 47.53 mg/kg of malvidin were detected (Table 1).

### 3.2. Hepatoprotective Effects of AM

Compared with normal control mice, a 24-week course of alcohol consumption led to a 31.2% decrease in body weight. Meanwhile, both AM and Sil administration prevented this reduction in body weight (*p* < 0.05; [Supplementary-material supplementary-material-1]). Compared with the model mice, AM strongly suppressed swelling of the liver (*p* < 0.05), kidney (*p* < 0.01), and heart (*p* < 0.01) but had no effect on the spleen ([Supplementary-material supplementary-material-1]). Sil only reduced liver swelling in mice with chronic alcohol-induced damage (*p* < 0.05), but not the swelling of other organs ([Supplementary-material supplementary-material-1]).

**Figure 2:**
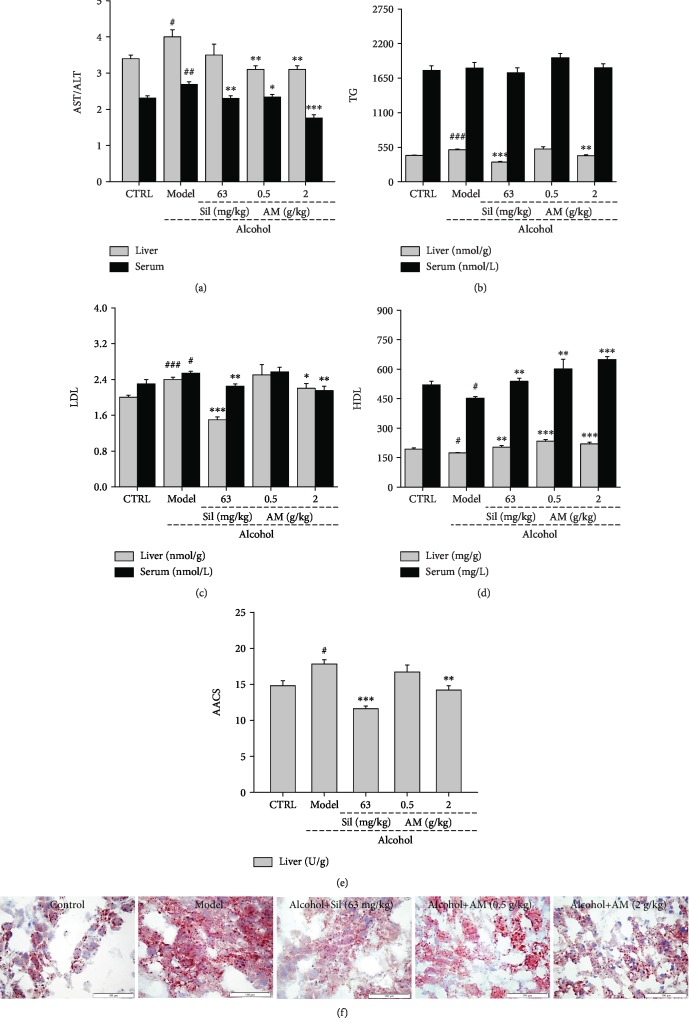
The antisteatosis effects of AM in mice with chronic alcohol damage. AM and Sil reduced (a) the ratio of AST/ALT and the levels of (b) TG and (c) LDL, (d) increased the levels of HDL in the serum and liver, and (e) reduced the levels of AACS in the serum of mice with chronic alcohol-induced liver injury. (f) The lipid accumulation alternations in the liver of mice with chronic alcohol injury were strongly reversed after AM and Sil administration (magnification: 400x, scale bar: 100 *μ*m). AM: *Aronia melanocarpa*; Sil: silybin. The data were analyzed using a one-way ANOVA and expressed as means ± *S*.*E*.*M*. (*n* = 10). ^#^*p* < 0.05, ^##^*p* < 0.01, and ^###^*p* < 0.001 vs. the control group; ^∗^*p* < 0.05, ^∗∗^*p* < 0.01, and ^∗∗∗^*p* < 0.001 vs. the alcohol-only treated model group.

The serum AST/ALT levels served as biochemical markers of liver injury [34]. Compared with the normal control mice, alcohol administration strongly increased values of AST/ALT in both the serum and liver (*p* < 0.05), while AM administration prevented these increases (*p* < 0.05; Figure 2(a)).

Subsequently, the antisteatosis effects of AM were further analyzed in mice with chronic alcohol-induced damage. AM increased the HDL levels > 33.0% (*p* < 0.01) (Figure 2(d)) and reduced the LDL levels by 14.0% (*p* < 0.01 Figure 2(c)) in the serum of 6-month alcohol-exposed mice. Neither AM nor Sil had a significant effect on the serum TG level (Figure 2(b)). In the livers of alcohol-exposed mice, AM administration resulted in 18.5%, 8.3%, and 20.2% reductions in the levels of TG (*p* < 0.01; Figure 2(b)), LDL (*p* < 0.05; Figure 2(c)), and AACS (*p* < 0.01; Figure 2(e)), respectively, and a >26.3% increase in the level of HDL (*p* < 0.001; Figure 2(d)). Sil strongly prevented the increases in the levels of TG (*p* < 0.001; Figure 2(b)) and LDL (*p* < 0.001; Figure 2(c)) and elevated levels of HDL (*p* < 0.01) in the liver (Figure 2(d)). The Oil red O staining further confirmed the protection of AM on the liver indicated by the reduced numbers of lipid droplets in the liver of chronic alcohol-damaged mice after AM and Sil administration (Figure 2(e)).

**Figure 3:**
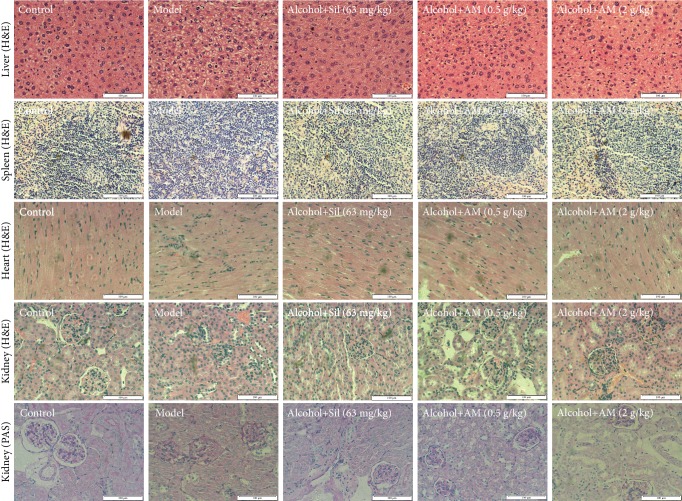
The pathological alternations in the liver, spleen, heart, and kidney of mice with chronic alcohol injury were strongly reversed after AM and Sil administration (magnification: 400x, scale bar: 100 *μ*m). AM: *Aronia melanocarpa*; Sil: silybin.

Both AM and Sil strongly protected the liver against chronic alcohol damage, suggesting that these reagents alleviated the formation of lipid droplets and punctate necrosis and reduced the infiltration of inflammatory factors (Figure 3). In the kidney, excessive alcohol caused narrowing of the glomerular capsule and hypertrophy of glomerular cells, both of which were significantly alleviated by AM and Sil as determined in H&E-stained sections (Figure 3). PAS staining further confirmed that Sil and AM prevented kidney inflammation in chronic alcohol-damaged mice, as indicated by a reduction in PAS-positive areas (Figure 3). Furthermore, AM and Sil suppressed the inflammatory infiltration into the hearts of mice subjected to chronic alcohol consumption.

### 3.3. Anti-Inflammatory Effects of AM

ILs, particularly IL-2, IL-4, and IL-6, are involved in systemic inflammatory responses and can induce liver cell damage via negative feedback pathways [35]. THEM4, an inhibitor of Akt, can effectively decrease the phosphorylation of Akt in the liver [36]. The levels of FGL1 and FGG, two acute-phase reactants, increase with the upregulation of IL-6 during inflammatory responses [37, 38]. AM and Sil significantly prevented increases in the hepatic levels of IL-2 (*p* < 0.05), IL-4 (*p* < 0.05), IL-6 (*p* < 0.05), THEM4 (*p* < 0.05), FGL1 (*p* < 0.05), and FGG (*p* < 0.01) in mice with chronic alcohol damage (Table 2).

### 3.4. Antioxidative Effects of AM

Oxidative stress, which is caused by free radicals produced in response to alcohol consumption, has been reported as an important factor in the progression of liver disease [39]. Coenzyme Q3, CAT, and SOD are effective free radical scavengers and antioxidants, while MGST3 can effectively isolate harmful lipophilic compounds. Compared with mice exposed only to alcohol, AM prevented increases in the levels of MDA (*p* < 0.05), ROS (*p* < 0.05), APCS (*p* < 0.01), and CYP8B1 (*p* < 0.01) and reductions in the levels of CAT (*p* < 0.01), SOD (*p* < 0.05), GSH-Px (*p* < 0.05), CoQ3 (*p* < 0.05), and MGST3 (*p* < 0.001) in the livers of mice with chronic alcohol damage (Table 3). Sil exhibited similar antioxidative effects but failed to influence the hepatic levels of CoQ3 (Table 3).

### 3.5. Regulatory Effects of AM on Nrf2 Signaling

**Figure 4:**
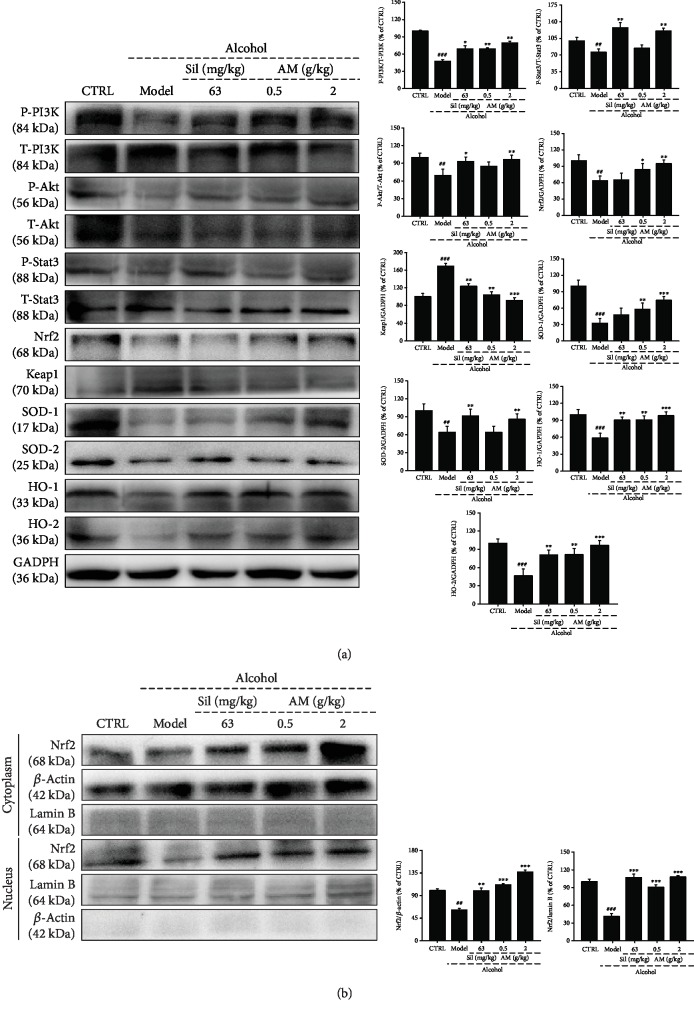
Nrf2 signaling regulated by PI3K/Akt activation is involved in AM-mediated liver protection in mice with chronic alcohol injury. (a) AM and Sil increased the expression levels of P-PI3K, P-Akt, P-Stat3, Nrf2, SOD-1/2, and HO-1/2 in livers. (b) AM and Sil increased the expression levels of Nrf2 in the cytoplasm and nucleus of liver tissues. The data of quantified protein expressions were normalized by related total protein expressions, GAPDH, *β*-actin, and/or lamin B. The data were analyzed using one-way ANOVA and expressed as means ± S.E.M. (*n* = 4). ^##^*p* < 0.01 and ^###^*p* < 0.001 vs. the control group; ^∗^*p* < 0.05, ^∗∗^*p* < 0.01, and ^∗∗∗^*p* < 0.001 vs. the alcohol-only treated model group. AM: *Aronia melanocarpa*; Sil: silybin.

PI3K/Akt signaling plays a critical role in the regulation of lipid metabolism and hepatocyte apoptosis in the liver and is regulated via a feedback loop with the Nrf2 signaling pathway [40]. Both AM and Sil enhanced the phosphorylation levels of PI3K (*p* < 0.05), Akt (*p* < 0.05), and STAT3 (*p* < 0.01) in the liver of mice with 6-month alcohol consumption (Figure 4(a)). Nrf2 signaling is involved in liver inflammation induced by long-term alcohol intake [41]. Both AM and Sil prevented the overexpression of Keap1 and reversed the low expression levels of Nrf2 (*p* < 0.05) and its downstream effectors, including HO-1 (*p* < 0.01), HO-2 (*p* < 0.01), SOD-1 (*p* < 0.01), and SOD-2 (*p* < 0.01) in the livers of alcohol-injured mice (Figure 4(a)).

The low expression levels of Nrf2 in both the cytoplasm and nucleus were noted in the liver of long-term alcohol intake mice (*p* < 0.001), which were all strongly enhanced by AM and Sil (*p* < 0.001; Figure 4(b)).

## 4. Discussion

The nutritive value of AM was indicated by an analysis of its contents, which include 18 types of fatty acids, 17 types of amino acids, 4 types of anthocyanidins (including delphinidin and cyanidin), and 14 types of minerals. Previous studies identified anthocyanins among the most effective antioxidants and free radical scavengers, with various pharmacological efficacies [42]. Additionally, anthocyanin pretreatment significantly inhibits the alcohol-induced depletion of hepatic GSH and SOD [43]. AM is composed of approximately 0.32% anthocyanins, which provide a basis for the hepatoprotective properties of this product against alcohol-induced oxidative and inflammatory liver injury.

In a mouse model of chronic alcoholic liver injury, AM effectively restored the AST/ALT ratio and alleviated pathological damage to the organs, particularly the liver. These findings from our study confirm the hepatoprotective effects of AM during chronic alcohol intake. During moderate or severe hepatitis, AST is released consequent to hepatocyte mitochondrial damage, leading to an increased AST/ALT ratio. This phenomenon is a hallmark of early liver injury [34]. AACS, as a lipid metabolism factor, helps to synthesize fatty acids and cholesterol, highly related with fat metabolism dysfunction [44]. Fat metabolism dysfunction is considered to be the progression of alcoholic fatty liver, which can further induce oxidative stress and inflammatory response [6, 45]. AM effectively modulates the levels of AACS and reduces the numbers of lipid droplets in the liver, suggesting its inhibition on fat metabolism disorders. During the process of long-term alcoholic liver damage, alcohol is oxidized predominantly to acetaldehyde by alcohol dehydrogenase in hepatocytes, and this process promotes the synthesis of fatty acids. Eventually, this process leads to fatty liver and fat metabolism disorders, which are considered important processes in ALD and are closely related to oxidative stress [46]. AM effectively modulates the levels of factors associated with lipid metabolism, suggesting an ability of this product to prevent fat metabolism disorders and protect the liver. Metabolites such as ROS and acetaldehyde, which are generated during long-term alcohol consumption, are responsible for the inflammatory response in the liver. The levels of fibrinogens (e.g., FGL1 and FGG) are increased in most patients with severe liver disease [47]. An increased level of IL-2 is associated with hepatic fibrosis in humans with ALD [48]. A causal link has been established between aberrant levels of IL-4 and IL-6 and alcohol-induced hepatitis, primary biliary cirrhosis, and chronic hepatitis in humans [49, 50]. This study has successfully confirmed the anti-inflammatory effects of AM in mice with long-term alcohol damage.

Oxidative stress is a well-known cause of alcohol-induced liver injury, particularly as the liver is poorly resistant to excessive ROS [51]. ROS are responsible for inducing the inflammatory infiltration of neutrophils and the apoptosis and eventual necrosis of hepatocytes [52]. ROS is also known to affect lipid peroxidation [53]. An increase in the hepatic level of MDA is consistent with dyslipidemia, and reflects the degree of lipid peroxidation and the extent of hepatocyte damage [54]. As effective antioxidant enzymes, O^2-^ and H_2_O_2_ could be converted to H_2_O by SOD and CAT inside cells [55], which has been reported as the first line of defense against ROS overaccumulation [56]. Furthermore, MGST3 participates in the detoxification process and effectively isolates harmful lipophilic compounds such as alcohol [57], thus protecting cells by preventing the oxidative destruction of sulfhydryl proteins [58, 59]. CoQ3, one of the most effective free radical scavengers, helps to suppress endogenous free fatty acids [60], which are present at 10-fold higher levels in ALD patients than in healthy people [61]. CYP8B1 is required for the synthesis of cholic acid [62] that stimulates mitochondria to produce excess ROS in liver [63]. In this study, AM was observed to exert antioxidative effects by regulating various anti- and prooxidative factors in mice with alcohol-induced chronic liver injury. The overaccumulation of ROS in response to alcohol metabolism is sufficient to promote cellular necrosis, which in turn activates inflammatory responses and amplifies inflammatory signaling processes [64]. The activation of T cells by ROS induces the release of inflammatory factors, including IL-2, IL-4, and IL-6 [65]. Our data suggest that the hepatoprotective effects of AM against chronic alcohol damage are related to its excellent antioxidative properties.

As a key transcription factor regulating oxidative damage, Nrf2 is regulated by ROS accumulation during alcohol administration. ROS modifies the thiol group in the intervention region (IVR) of Keap1, causing a conformational change in Keap1, resulting in its dissociation with Nrf2 or reduced ubiquitination of Nrf2, which then enter the nucleus to play an antioxidative role via regulating its downstream antioxidant enzymes, including SOD, CAT, and HO-1 [66]. STAT3, a key mediator of anti-inflammatory cytokines, is involved in Nrf2-medieated cytoprotection, which can be regulated by HO-1 [67]. The phosphorylated STAT3 can trigger the activation of PI3K/Akt signaling further silencing the increased phosphorylation levels of NF-*κ*B and suppressing its downstream proinflammatory gene programs [68]. According to the previous study, Nrf2 can be regulated by PI3K/Akt signaling [69]. The pretreatment of PI3K inhibitor, LY294002, significantly inhibits the activation of Nrf2 [69]. The antioxidative property of AM is related to its modulation on Nrf2 signaling, which is further regulated by the phosphorylation activities of PI3K/Akt.

There are still limits in this study. Based on the present data, we failed to conclude the main active ingredients contained in AM that show the protection on the liver against long-term alcohol exposure. Although the acute toxicity test has been performed in the preliminary experiment, which suggested the safe usage of AM on mice, more experiments should be applied to confirm its safety. Furthermore, during this study, the spleen and heart were found to have different degrees of damage. These injuries were related to long-term alcohol intake, and AM had protective effects on these organs. However, there is no in-depth report in this topic; we will conduct follow-up research.

Taken together, this study first confirmed that 6-week AM administration can alleviate liver damage in mice induced by 24-week alcohol feeding through inhibition of oxidative stress and reduction of inflammatory reactions. The possible mechanism of AM's liver protection may be related to its regulation on Nrf2 signaling via the activities of PI3K/Akt in C57BL/6 mice with alcohol-induced chronic liver injury.

## Figures and Tables

**Table 1:** Composition of *Aronia melanocarpa* (AM).

	Compounds	Content	Compounds	Content	Compounds	Content
Main components (g/100 g)	Total sugar	75.96	Reducing sugar	29.3	Protein	6.61
	Total ash	4.03	Total alkaloid	0.065	Total flavonoids	0.021

Anthocyanidin (mg/kg)	Delphinidin	24.43	Cyanidin	3140.45	Petunia pigment	*ND*
	Pelargonidin	*ND*	Peonidin	*ND*	Malvidin	47.53

Nucleotide (mg/100 g)	CMP	3.21	AMP	6.51	GMP	*ND*
	IMP	*ND*	UMP	1.25		

Fatty acid (g/100 g)	Capric acid (C10:0)	0.021	Stearic acid (C18:0)	0.26	Arachidonic acid (C20:4n6)	*ND*
	Undecanoic acid (C11:0)	*ND*	Oleic acid (C18:1n9c)	2.19	Eicosapentaenoic acid (C20:5n3)	0.022
	Lauric acid (C12:0)	0.032	Elaidic acid (C18:1n9t)	*ND*	Heneicosanoic acid (C21:0)	*ND*
	Tridecanoic acid (C13:0)	*ND*	Linoleic acid (C18:2n6c)	4.62	Docosanoic acid (C22:0)	0.023
	Myristic acid (C14:0)	0.041	Translinoleic acid (C18:2n6t)	*ND*	Erucic acid (C22:1n9)	0.37
	Myristoleic acid (C14:1)	*ND*	*α*-Linolenic acid (C18:3n3)	*ND*	*cis*-13,16-Docosadienoic acid methyl ester (C22:2)	*ND*
	Pentadecanoic acid (C15:0)	0.0088	*γ*-Linolenic acid (C18:3n6)	*ND*	Docosahexaenoic acid (C22:6n3)	*ND*
	Pentadecenoic acid (C15:1)	0.031	Arachidic acid (C20:0)	0.079	Tricosanoic acid (C23:0)	0.058
	Hexadecanoic acid (C16:0)	1.04	Paullinic acid (C20:1)	0.32	Tetracosanoic acid (C24:0)	*ND*
	Palmitoleic acid (C16:1)	0.11	Eicosadienoic acid (C20:2)	*ND*	Nervonic acid (C24:1n9)	*ND*
	Heptadecanoic acid (C17:0)	0.027	Eicosatrienoic acid (C20:3n3)	*ND*	Octanoic acid (C8:0)	*ND*
	Ginkgolic acid (C17:1)	*ND*	Dihomo-gamma-linolenic acid (C20:3n6)	0.18		

Amino acid (g/kg)	Aspartic acid (asp)	11.46	Cystine (Cys)	0.48	Phenylalanine (Phe)	1.94
	L-Threonine (Thr)	2.64	Valine (Val)	2.20	Lysine (Lys)	1.69
	Serine (Ser)	2.83	Methionine (Met)	1.20	Histidine (His)	1.94
	Glutamic acid (Glu)	5.34	Isoleucine (Iso)	2.01	Arginine (Arg)	5.26
	Glycine (Gly)	2.89	Leucine (Leu)	3.22	Proline (Pro)	8.60
	Alanine (ala)	1.54	Tyrosine (Tyr)	1.48		

Minerals (*μ*g/kg)	Mercury (hg)	160.00	Zinc (Zn)	28.17	Cuprum (Cu)	3634.39
	Lead (Pb)	400.98	Ferrum (Fe)	124.1	Natrium (Na)	101.0
	Selenium (Se)	80.49	Manganese (Mn)	28.30	Kalium (K)	1.56
	Arsenic (As)	93.66	Chromium (Cr)	629.51	Magnesium (Mg)	1324
	Cadmium (Cd)	298.29	Calcium (Ca)	2281		

ND: not detected.

**Table 2:** The effects of AM on inflammatory factors in liver of mice with chronic alcohol injury.

	CTRL	Alcohol			
		Model	Sil (63 mg/kg)	AM (0.5 g/kg)	AM (2 g/kg)
IL-2 (pg/mg)	42.4 ± 1.9	49.6 ± 1.5^#^	43.6 ± 1^∗^	50.9 ± 1.2	43.8 ± 0.4^∗^
IL-4 (pg/mg)	9.7 ± 1.2	12.9 ± 0.5^#^	9.5±1.1^∗∗^	11.2 ± 0.3	10.2 ± 0.7^∗^
IL-6 (pg/mg)	17.4 ± 0.5	17.4 ± 0.4	15.2 ± 0.4^∗^	16.5 ± 0.2	14.8 ± 0.5^∗^
THEM4 (pg/mg)	159.5 ± 8.3	204.2 ± 8.1^##^	112.5±6.5^∗∗∗^	175.9 ± 7.8	174.2 ± 5.4^∗^
FGL1 (ng/mg)	1.4 ± 0.06	1.7 ± 0.07^#^	1.1±0.04^∗∗∗^	1.6 ± 0.08	1.4 ± 0.03^∗^
FGG (*μ*g/mg)	129 ± 4.5	153.6 ± 4.5^##^	94.7±3.6^∗∗∗^	164.4 ± 9.1	126.5±7.4^∗∗^

All date are presented as mean ± S.E.M. (*n* = 10). ^#^*p* < 0.05 and ^##^*p* < 0.01 compared with the control group; ^∗^*p* < 0.05, ^∗∗^*p* < 0.01, and ^∗∗∗^*p* < 0.001 compared with the alcohol-only treated model group.

**Table 3:** The effects of AM on pro- and antioxidation factors in the liver of mice with chronic alcohol injury.

	CTRL	Alcohol			
		Model	Sil (63 mg/kg)	AM (0.5 g/kg)	AM (2 g/kg)
MDA (nmol/mg)	3.9 ± 0.15	4.5 ± 0.18^#^	3.6 ± 0.19^∗^	3.8 ± 0.11^∗^	3.7±0.04^∗∗^
ROS (U/mg)	32.1 ± 1.5	42.7 ± 1.3^##^	35.1±1.1^∗∗^	35.3 ± 1.6^∗^	33.1±0.7^∗∗^
CAT (U/mg)	0.39 ± 0.09	0.15 ± 0.04^###^	0.21±0.08^∗∗^	0.19±0.02^∗∗^	0.31±0.03^∗∗∗^
SOD (U/mg)	9.0 ± 1.3	4.1 ± 0.4^###^	5.8 ± 0.3^∗^	5.4 ± 0.3^∗^	7.6±0.7^∗∗^
GSH-PX (U/mg)	15.9 ± 1.7	7.9 ± 1.1^###^	18.8±2.0^∗∗∗^	11.6 ± 0.7^∗^	20.1±1.9^∗∗∗^
CoQ3 (U/g)	16.9 ± 0.5	15.1 ± 0.6^#^	15.6 ± 0.7	17.2 ± 0.8^∗^	18.3±0.3^∗∗^
MGST3 (U/g)	115.2 ± 2.1	103.4 ± 1.8^#^	111.4 ± 2.8^∗^	124.7±1.9^∗∗∗^	125.1±4.1^∗∗∗^
APCS (ng/mg)	123.8 ± 4.5	150.2 ± 6.2^##^	95.4±2.9^∗∗∗^	162.8 ± 4.4	115.5±3.6^∗∗^
CYP8B1 (ng/mg)	1.21 ± 0.04	1.43 ± 0.05^##^	0.93±0.02^∗∗∗^	1.43 ± 0.05	1.14±0.04^∗∗^

All date are presented as mean ± S.E.M. (*n* = 10). ^#^*p* < 0.05, ^##^*p* < 0.01, and ^###^*p* < 0.001 compared with the control group; ^∗^*p* < 0.05, ^∗∗^*p* < 0.01, and ^∗∗∗^*p* < 0.001 compared with the alcohol-only treated model group.

## Data Availability

All generated and analyzed data used to support the findings of this study are included within the article.

## Supplementary Materials

Supplementary Materials Table S1: the effects of AM treatment on the body weights and organ indexes of mice with chronic alcohol injury.
