# Supplementary material for: Aronia melanocarpa Prevents Alcohol-Induced Chronic Liver Injury via Regulation of Nrf2 Signaling in C57BL/6 Mice
Source: Oxid Med Cell Longev. 2020 Jan 8;2020:4054520. doi: 10.1155/2020/4054520 (PMC6970495; doi:10.1155/2020/4054520)
Supplement: Supplementary Materials — Table S1: the effects of AM treatment on the body weights and organ indexes of mice with chronic alcohol injury. [file 4054520.f1.docx]

Supplemental Data

**Table S1** The effects of AM treatment on the body weights and organ indexes of mice with chronic alcohol injury

|  |  | CTRL | Alcohol | | | |
| --- | --- | --- | --- | --- | --- | --- |
|  |  |  | Model | Sil (63 mg/kg) | AM (0.5 g/kg) | AM (2 g/kg) |
| Body weight (g) (weekly) | 1^st^ | 24.7±0.3 | 23.7±0.4^#^ | 24.1±0.3 | 24.3±0.4 | 24±0.5 |
|  | 5^th^ | 27.3±0.4 | 24.5±0.5^#^ | 24.4±0.6 | 22.7±0.3 | 23.6±0.6 |
|  | 10^th^ | 28.5±0.4 | 26.9±0.5^#^ | 26.5±0.7 | 26.3±0.4 | 26.7±0.7 |
|  | 19^th^ | 29±0.4 | 26.4±0.5^#^ | 26.9±0.7 | 27.3±0.3 | 25.3±0.4 |
|  | 20^th^ | 31.9±0.4 | 26.6±0.5^##^ | 26.7±0.5 | 27.6±0.5 | 26.5±0.5 |
|  | 21^th^ | 31.7±0.4 | 25.7±0.3^##^ | 26.3±0.4 | 24.6±0.3 | 25.1±0.5 |
|  | 22^th^ | 30.9±0.3 | 23.4±0.4^###^ | 25.3±0.3^*^ | 24.3±0.4 | 24.7±0.4^*^ |
|  | 23^th^ | 31.3±0.3 | 22.4±0.5^###^ | 24.3±0.4^*^ | 23.3±0.7 | 24.5±0.5^*^ |
|  | 24^th^ | 32.4±0.3 | 22.3±0.5^###^ | 24.6±0.4^*^ | 22.8±1.2 | 25.1±0.6^*^ |
| Organ Index  (g/100 g body weight) | Liver | 4.03±0.08 | 4.71±0.06^##^ | 4.28±0.08^*^ | 4.16±0.14^*^ | 4.41±0.12^*^ |
|  | Kidney | 1.27±0.03 | 1.53±0.02^###^ | 1.47±0.03 | 1.32±0.07^**^ | 1.46±0.04 |
|  | Spleen | 0.31±0.01 | 0.31±0.02 | 0.27±0.02 | 0.26±0.01 | 0.26±0.01 |
|  | Heart | 0.48±0.01 | 0.57±0.01^##^ | 0.53±0.02 | 0.49±0.02^**^ | 0.47±0.01^**^ |

All date is presented as mean ± S.E.M. (n=10). ^#^ *p* < 0.05, ^##^*p* < 0.01 and ^###^ *p* < 0.001 compared with control group; ^*^ *p* < 0.05 and ^**^ *p* < 0.01 compared with alcohol-only treated model group. AM: *Aronia melanocarpa*; Sil: silybin.
